# Impact of prophylactic central lymph node dissection on the complications and recurrence rates in papillary thyroid carcinoma—An AFCE (French‐speaking Association of Endocrine Surgery) multicentre study based on the EUROCRINE® national data

**DOI:** 10.1002/cnr2.1993

**Published:** 2024-02-13

**Authors:** Nathalie Chereau, Niki Christou, Robert Caiazzo, Adrien Le Fouler, Jean Christophe Lifante, Laure Maillard, Eric Mirallie, Francois Pattou, Nicolas Bouviez, Nicolas Santucci, Laurent Brunaud, Fabrice Menegaux

**Affiliations:** ^1^ Pitié Salpêtrière Hospital, APHP Sorbonne University Paris France; ^2^ University Hospital Limoges Limoges France; ^3^ Claude Huriez Hospital Lille France; ^4^ Avicenne, APHP Bobigny France; ^5^ Lyon Sud Lyon France; ^6^ Chirurgie Cancérologique, Digestive et Endocrinienne, Institut des Maladies de l'Appareil Digestif Nantes University Nantes France; ^7^ University Hospital of Besançon Besancon France; ^8^ Dijon University Hospital Dijon France; ^9^ Brabois Hospital Vandoeuvre‐les‐Nancy France

**Keywords:** hypocalcemia, lymph node dissections, papillary thyroid carcinoma, recurrence, TNM staging

## Abstract

**Background:**

Prophylactic central neck dissection (pCND) remains controversial during the initial surgery for preoperative and intraoperative node‐negative (cN0) papillary thyroid carcinoma (PTC).

**Methods:**

Patients undergoing thyroidectomy with or without pCND (Nx) for PTC in nine French surgical departments, registered in the EUROCRINE® national data in France between January 2015 and June 2021, were included in a cohort study. Demographic and clinicopathological characteristics, complications, and recurrence rates were compared using multivariate regression analysis.

**Results:**

A total of 1905 patients with cN0 PTC were enrolled, including 1534 who had undergone pCND and 371 who hadn't (Nx). Of these, 1546 (81.2%) were female, and the median age was 49 years (range: 15–89 years). Patients who had undergone pCND were more likely to have multifocal tumors (*n* = 524 [34.2%] vs. *n* = 68 [18.3%], *p* < .001) and larger tumors (15.3 vs. 10.2 mm, *p* = .01) than patients with Nx. Of the patients with pCND, 553 (36%) had positive central LN (N1a), with a median of 1 N1 (IQR 0–5). pCND was associated with a higher temporary hypocalcemia rate (*n* = 25 [8%] vs. *n* = 15 [4%], *p* < .001). The rates of permanent hypocalcemia and temporary and permanent recurrent laryngeal nerve (RLN) palsy were not significantly different between the two groups (*p* > .2). After adjusting for covariates (age, sex, multifocality, and pathological T stage) in a multivariable Cox PH model, the performance of lymph node dissection (pCND vs. no‐pCND) was not associated with PTC recurrence (*p* = .2).

**Conclusion:**

pCND in PTC does not reduce recurrence and is associated with a two‐fold increase in the incidence of transient hypoparathyroidism. These data should be considered while issuing further guidelines regarding the treatment of patients with cN0 PTC.

## 1 INTRODUCTION

The performance of prophylactic central neck dissection (pCND) at the time of total thyroidectomy (TT) in clinically node‐negative (cN0) papillary thyroid carcinoma (PTC) remains controversial. Although occult central lymph node metastases are common, it is unclear whether the removal of these metastases initially would reduce the risk of recurrence. The data interpretation depends on the weight assigned to the risks and benefits of pCND. According to the American Thyroid Association (ATA) guidelines, the risks due to pCND outweigh the potential benefits, and CND should be limited to patients with preoperative central node positive (cN1) or cN0 who have advanced primary tumors (stage T‐TNM T3 or T4) or clinically involved lateral neck nodes cN1b.^1^ High‐level evidence supporting a lower risk of recurrent disease after pCND is lacking.^2^, ^3^, ^4^ Other authors have suggested that the primary benefit of pCND for cN0‐PTC is the detection of occult lymph node metastases, which may improve the accuracy of the nodal staging of cancer for postoperative management with regard to radioactive iodine administration (RAI).^5^, ^6^, ^7^ Although it is not evident that pCND improves patient prognosis, some authors recognize the benefit of pCND in limiting the need for a redo surgery for recurrence in the central compartment, which has a risk of recurrent laryngeal nerve injury and permanent hypoparathyroidism.^8^

This study aimed to compare the postoperative complications (hypocalcemia and RLN palsy) and outcomes (recurrence rates) between the cN0 PTC patients who underwent TT with pCND and those who underwent TT alone (Nx) in nine French departments of endocrine surgery, where pCND was performed by experienced endocrine surgeons.

## 2 MATERIALS AND METHODS

pCND has been defined by the American Society of Head and Neck Surgery and successively by ATA as a comprehensive, compartment‐oriented removal of the prelaryngeal (along the recurrent nerve) and pretracheal lymph nodes even if nodal metastasis is not detected clinically or radiologically before or during surgery. The data of adult patients with PTC who underwent total thyroidectomy (TT) with or without pCND (patients who underwent TT for a non‐malignant disease but were then diagnosed with PTC on final pathology) in nine French departments of endocrine surgery from January 2015 to June 2021 were retrieved from the prospectively maintained EUROCRINE©database and retrospectively analyzed. pCND was performed in all patients who were preoperatively (via ultrasound‐guided fine‐needle aspiration cytology) cN0 and/or intraoperatively (via intraoperative frozen section) classified as pN0.

This particular cohort of patients lends itself to this study because it represents a homogeneous group of cN0 patients who underwent a standardized surgical procedure: TT and systematic pCND, thus eliminating the variability in surgical techniques and approaches. Since all of our patients had a central neck dissection regardless of preoperative staging, this dataset is unique and can answer questions for the vast majority of surgeons who do not perform pCND.

Patients with therapeutic LND (Lymph Node Dissection), unilateral thyroid resection with pCND, and distant metastatic stage cancer M1 were excluded.

### 2.1 Radioactive iodine (RAI) therapy

The RAI dose was determined according to the American Thyroid Association (ATA) guidelines and the French Societies of Nuclear Medicine and Endocrinology. Patients at a high risk of recurrence (based on the ATA risk stratification^1^) received 100 mCi of RAI after thyroid hormone withdrawal for 4 weeks. Patients with intermediate‐risk PTC received 30 or 100 mCi of RAI and were prepared with recombinant human TSH prior to RAI administration (including pN1a with LN >0.2 cm). Patients with low‐risk PTCs were managed without RAI treatment.

### 2.2 Complications

Postoperative hypocalcemia was defined as a serum calcium level of less than 8 mg/dL (normal range: 8.4–10.6 mg/dL) after TT that was symptomatic with overt manifestations (such as anxiety, carpopedal spasms, tingling, or numbness of the extremities).

Hypocalcemia was considered permanent if the plasma parathyroid hormone (PTH) levels 6 months postoperatively were less than 15 pg/mL (normal range: 15–65 pg/mL), and the patient continued to require oral calcium (calcium carbonate) and vitamin D supplementation, in addition to the supplements that were taken routinely before thyroidectomy.

During the postoperative course, indirect laryngoscopy was performed in patients with dyspnea, hoarseness, or loss of voice quality. When RLN palsy was diagnosed a trial of speech therapy was initiated during the first week and continued for several months. RLN palsy was considered permanent if there was no proof of recovery using laryngoscopy within 6 months of the operation.

### 2.3 Follow‐up and recurrence

All patients followed a standardized surveillance schedule, which included physical examination, neck ultrasound, serum thyroglobulin (*T*_g_), and *T*_g_ autoantibody measurements after stimulation or under suppressive treatment at 6 and 12 months and annually thereafter. For patients who appeared to be disease‐free after 6 years of follow‐up, three‐yearly correspondence was established with them or their referring physicians. None of the patients were lost to follow‐up.

Recurrent disease was determined according to the French Society of Nuclear Medicine and Endocrinology guidelines and was defined as follows:

- Presence of suspicious lymph node (LN) in the central or lateral part of the neck or abnormal tissue in the thyroid bed with positive fine‐needle aspiration biopsy (locoregional recurrence).
- Biochemical evidence of disease: stimulated *T*_g_ level >10 ng/mL after thyroid hormone withdrawal or 5 ng/mL after recombinant human TSH administration; progression of basal (unsuppressed) *T*_g_ and/or basal *T*_g_ >1 ng/mL using the same assay technique; appearance or progressive elevation (>50%) of *T*_g_ autoantibodies using the same assay technique; and/or isolated and repeatedly elevated serum *T*_g_ levels.

### 2.4 EUROCRINE® database

EUROCRINE® is an online prospective database that has registered endocrine surgical procedures in centers across Europe since 2015. EUROCRINE® was started as a project within the Health Programme of the European Union in 2013 to reduce morbidity and mortality in patients undergoing surgical treatment for endocrine tumors. EUROCRINE® is managed by the EUROCRINE® Society, based in Vienna, and with a council of 13 participating national endocrine surgical societies and the European Society of Endocrine Surgeons. Personal registration is needed to enter the data in EUROCRINE®, which was supplied to the principal investigator at each participating center. Data collection was supervised by the principal surgeon at each site. The EUROCRINE® database is currently used by 310 departments and clinics in Europe, including 12 in France (https://www.eurocrine.eu). The platform is owned by Region Skåne, Sweden. Participating centers obtained informed consent from individual patients, and the pseudonymized data were stored as per the General Data Protection Regulation. This study was approved by the Ethical Committee of Lund University and Council and Steering Committee of EUROCRINE®.

### 2.5 Statistical analysis

Adult patients with PTC who underwent TT with or without pCND in a French cohort were selected for this study. Demographic and clinicopathological characteristics and complication rates were compared between the two groups using the Kruskal‐Wallis test for continuous variables and the *χ* ^2^ test for categorical variables. The odds of complications were compared, between patients with underwent pCND versus those who did not, using multivariable logistic regression analysis.

The multivariable Cox proportional hazards model was used to examine factors associated with the likelihood of PTC recurrence in the entire cohort. Only type of surgery (pCND or no pCND) age, sex, tumor multifocality, and pathological stage T‐TNM were adjusted for, owing to the limited number of events in this subgroup of patients.

A two‐sided significance level of .05 was used for all statistical tests. Analyses were performed using SAS v. 9.4 (SAS Institute, Cary, NC, USA). This study was approved by the Sorbonne University Hospital with an institutional review board exemption.

## 3 RESULTS

### 3.1 Patient characteristics

A total of 1905 cN0 PTC patients underwent total thyroidectomy with a median follow‐up period of 3 years. Of these, 1534 patients (80.5%) underwent pCND while 371 did not, usually PTC was diagnosed on final pathology. Overall, 81.2% of the patients in our cohort were women, and the median age was 49 years (IQR = 36–58 years). pCND patients were more likely to have multifocal tumors (524 [34.2%] vs. 68 [18.3%], *p* < .001) and larger median tumor size (15.3 vs. 10.2 mm, *p* = .01) than no‐LND patients (Table 1). The median number of LN removed in the pCND group was 13 (IQR = 7–20). A total of 553 (36%) patients had positive LN (N1), with a median of 1 N1 (IQR 0–5).

**TABLE 1:** Demographic and clinicopathologic characteristics of patients with PTC who underwent thyroidectomy with or without pCND.

	Total (*N* = 1905)	Surgery type		*p*‐value
		pCND (*N* = 1534)	No‐pCND (*N* = 371)	
Age at diagnosis (years)	49 (39–60)	48 (36–58)	54 (43–63)	<.001
Sex, Female	1546 (81.2)	1270 (81.2)	301 (81.1)	.967
Lymphocytic thyroiditis	187 (9.8)	163 (10.6)	24 (6.5)	.02
Multifocality	592 (31.2)	524 (34.2)	68 (18.3)	<.001
Size of the largest tumor (mm)	9.0 (4.0–15.0)	15.3 (8.0–20.0)	10.2 (2.0–9.0)	.01
Number of tumors	1.0 (1.0–2.0)	1.0 (1.0–2.0)	1.0 (1.0–2.0)	.98
Pathologic T‐TNM (8th edition)				<.001
T1	1608 (84.4)	1260 (82.1)	348 (93.4)	
T2	215 (11.3)	204 (13.3)	11 (3)	
T3	72 (3.8)	62 (4)	10 (2.7)	
T4	10 (0.5)	8 (0.5)	2 (0.1)	
Pathologic N1a	‐	553 (35)	0 (0.0)	
Number of removed LN		13.0 (7.0–20.0)		
Number of positive^a^ cLN		1.0 (0.0–5.0)		

*Note*: T‐TNM: Tumor status in AJCC/UICC classification 8th edition. Data are presented as *N* (%) or median (interquartile range).

Abbreviations: LN, lymph node; pCND, prophylactic central neck dissection; PTC, papillary thyroid cancer.

^a^ Indicates only patients with central nodal disease with pathologic confirmation after surgery.

### 3.2 Complication rates

Patients who underwent pCND were more likely to develop temporary hypocalcemia than those who did not pCND (125 [8%] vs. 15 [4%], *p* < .001). Permanent hypocalcemia, temporary and permanent recurrent laryngeal nerve (RLN) paresis and abscess were not significantly different between the two groups (*p* = .2) (Table 2).

**TABLE 2:** Complications following thyroid surgery with or without pCND.

Nb of patients	Total *N* = 1905	pCND *N* = 1534	No pCND *N* = 371	*p* value
Temporary hypocalcemia	140 (7.3)	125 (8.1)	15 (4)	<.001
Permanent hypocalcemia	51 (2.4)	43 (2.8)	8 (2.1)	.21
Temporary RLN paresis	48 (2.7)	38 (2.5)	10 (2.8)	.60
Permanent RLN paresis	12 (0.6)	9 (0.6)	3 (0.7)	.53
Hematoma	13 (0.7)	9 (0.6)	4 (1.1)	.04
Abscess	6 (0.3)	5 (0.3)	1 (0.3)	.33

*Note*: Data are presented as *N* (%).

Abbreviations: IQR, interquartile range; pCND, prophylactic lymph node dissection; RLN, recurrent laryngeal nerve.

After adjusting for known covariates (age, sex, tumor focality, pathological T stage), temporary surgical complications and temporary hypocalcemia were more likely to occur in patients who had undergone pCND than those who did not (OR [95% CI]: 1.46 (1.19–1.78) and 1.53 (1.25–1.88), respectively). Permanent surgical complications and temporary recurrent laryngeal paresis did not differ between the two groups (Table 3).

**TABLE 3:** Unadjusted and adjusted odds of complications for patients with PTC who had pCND compared to those who had not undergone pCND.

	Unadjusted		Adjusted^a^	
	OR (95% CI)	*p*‐value	OR (95% CI)	*p*‐value
Temporary surgical complications	1.43 (1.19–1.72)	.0002	1.46 (1.19–1.78)	.0003
Hypocalcemia	1.54 (1.28–1.85)	<.0001	1.53 (1.25–1.88)	<.0001
RLN palsy	0.90 (0.50–1.34)	.60	0.96 (0.62–1.51)	.86
Permanent surgical complications	1.18 (0.79–1.75)	.42	1.14 (0.74–1.75)	.57

Abbreviations: CI, confidence interval; OR, odds ratio; pCND, prophylactic central neck dissection; PTC, papillary thyroid cancer; RLN, recurrent laryngeal nerve.

^a^ Adjusted for age, sex, tumor multifocality, and pathological T stage.

### 3.3 Factors associated with recurrence

In a univariate analysis, patients with pCND were more likely to have PTC recurrence than those without pCND (4.9% vs. 2.4%, *p* < .001), and the time to recurrence was shorter in the pCND group than in the no‐pCND group (72 months vs. 96 months, *p* < .001).

RAI therapy was used in 931 (49%) of the 1905 patients: 74/371 patients (20%) who underwent TT without pCND versus 857/1534 (55%) who underwent TT with pCND (*p* < 0·001). Of the 857 patients, 189 (22%) underwent RAI because of pN1 with LN >0.2 cm.

After adjusting for other variables in the multivariable Cox PH model, the type of surgery (pCND vs. no‐pCND) was not associated with PTC recurrence (*p* = .21). Patients with multifocal tumors were more likely to have PTC recurrence than those without multifocal tumors [HR 95% CI: 1.91 (1.35–2.70), *p* = .002]. A higher pathologic T stage was also associated with an increased risk of PTC recurrence in these patients (Table 4).

**TABLE 4:** Multivariable analysis of factors associated with disease recurrence in patients with PTC undergoing surgery.

Variable	Entire cohort	
	HR (95% CI)	*p*‐value
Procedure: pCND versus no PND	1.30 (0.86–1.98)	.21
Age (per 1‐year increase)	1.00 (0.99–1.01)	.83
Sex female	0.76 (0.51–1.12)	.17
Tumor multifocality	1.91 (1.35–2.70)	.002
Pathologic T‐TNM (AJCC 8th edition)		<.0001
T2	2.31 (1.52–3.50)	
T3	4.43 (2.50–7.84)	
T4	7.05 (3.39–14.67)	

*Note*: References are: no pCND; Age; Male; Unifocal tumors; Pathologic T1; N0 excluded.

Abbreviations: CI, confidence interval; HR, hazard ratio; no pCND, prophylactic lymph node dissection.

## 4 DISCUSSION

There is inadequate and conflicting data regarding the use of pCND in the management of PTC. A prospective randomized controlled trial evaluating the role of pCND in cN0 PTC is not feasible. Given the low rates of both newly identified structural diseases and the morbidity after surgery for cN0 PTC, prohibitively large sample sizes should be required for sufficient statistical power to demonstrate significant differences in outcomes.^2^ Hughes et al. found that pCND did not reduce locoregional recurrence and was associated with a two‐fold increase in permanent hypoparathyroidism. Based on their interpretation of the data, the risks of pCND outweigh its potential benefits, and CND should be limited to patients with cN1 stage PTC and large tumors (T3‐T4).^9^ However, the effect on recurrence rates remain uncertain, and many people stand in line.^5^, ^6^, ^7^ Some teams advise routine pCND because it can identify positive LN in 30%–50% of patients and enable their reclassification. This approach is based on studies demonstrating that pCND lowers the risk of developing persistent or recurrent disease and facilitates accurate disease staging, which subsequently improves treatment and follow‐up.^10^ The detection of positive lymph nodes is associated with the administration of higher doses of RAI for postoperative ablation, decreased recurrence in patients undergoing pCND, and the need for reoperation. Studies have reported higher rates of hypoparathyroidism and RLN palsy with re‐operative surgery because of tumor recurrence and local invasion.^11^ Barczyński et al. previously showed that patients treated with pCND had significantly improved survival compared to a historical control group of patients treated with total thyroidectomy alone, with 10‐year disease‐specific survival improving from 92.5% to 98.0%.^12^ A significant criticism of this study was the increased use of radioactive iodine (RAI) in the pCND group owing to the information gained from lymph node staging, which may have affected disease‐specific survival. While potentially confounding the outcome data, it could be argued that improved outcomes would not have been obtained without this additional prognostic information, further highlighting the prophylactic as opposed to the purely therapeutic benefit of pCND. A clearly identifiable bias in this study is that RAI therapy was used significantly more frequently in patients with TT with pCND: 64.5% versus 28.0%. However, the rationale for this statement was not an increase in availability or more liberal use but to obtain information concerning positive LN identified in 30.2% of the surgical specimens following pCND, who was considered an indication for RAI therapy. Thus, pCND upstaged many tumors that would otherwise not have been referred for adjuvant RAI treatment. Based on this strategy, patients who underwent pCND had a higher chance of receiving treatment for subclinical micrometastases. Recent studies have suggested that approximately one‐third of patients who underwent pCND may have been upstaged.^6^, ^13^ The present study's results agree with this observation, as 35% of our cN0 PTC patients who underwent pCND had central LN metastases N1a found at diagnosis, and 22% of pCND received RAI therapy for N1a >0.2 cm. Bonnet et al. reported retrospective data suggesting that pCND resulted in increased use of RAI and, ultimately, in more favorable outcomes.^14^

Detection of central neck lymph node metastasis using preoperative ultrasound examination remains difficult. Preoperative ultrasound for the detection of lymph node metastasis has a high specificity (92%) and positive predictive value (81%–92%) but low sensitivity (51%–61%) and negative predictive value especially for central LN (63%–76%).^15^, ^16^

Based on our data, in contrast to other series,^17^, ^18^, ^19^ we can conclude that pCND can be safely performed with comparable permanent morbidity by experienced surgeons, as we included nine specialized endocrine surgery departments, performing at least 50 thyroid operations annually and with experience in central lymph node dissection.

This study had some limitations. It was a retrospective design, and some data were lacking in the pathologic reports (e.g., size of the largest N1 and capsular effraction), short‐term follow‐up, and localization (and management) of recurrence. These data were not available in the Eurocrine database for our study. The authors recognize that prophylactic ipsilateral pCND is not a widely accepted procedure, nor is it recommended by the ATA guidelines. Also, one of the biases is that patients in pCND group received radioactive Iodine because of positive LNs in central compartment and it reduces the risk of recurrence. However, this dataset is unique and can provide data that other studies cannot. We believe that the large size of this multicentric cohort, with standardized treatment and unchanged follow‐up over time, overcomes the retrospective nature of the study and provides useful information for the management of cN0 PTC patients.

## 5 CONCLUSION

Many patients with cN0 PTC from the AFCE department underwent pCND. Although 35% of N1 stage cases, these data suggest that pCND does not improve oncological outcomes and is associated with increased postoperative transient hypocalcemia. These data should be considered while issuing further guidelines regarding patients with cN0 PTC.

## AUTHOR CONTRIBUTIONS

*Study conception and design*: N. Chereau and F. Menegaux. *Acquisition of data*: N. Chereau, N. Christou, R. Caiazzo, A. Le Fouler, J. C. Lifante, L. Maillard, E. Mirallie, F. Pattou, N. Bouviez, N. Santucci, L. Brunaud, and F. Menegaux. *Analysis and interpretation of data*: N. Chereau and F. Menegaux. *Drafting of manuscript*: N. Chereau and F. Menegaux. *Critical revision of manuscript*: N. Chereau, N. Christou, R. Caiazzo, A. Le Fouler, J. C. Lifante, L. Maillard, E. Mirallie, F. Pattou, N. Bouviez, N. Santucci, L. Brunaud, and F. Menegaux.

## CONFLICT OF INTEREST STATEMENT

The authors have stated explicitly that there are no conflicts of interest in connection with this article.

## Data Availability

This is a multicentric nationwide retrospective cohort study using the Eurocrine® Registry (www.eurocrine.eu). Eurocrine® is an endocrine surgical quality registry. Data are registered according to predefined data fields. They include preoperative settings, diagnostic work‐up, surgery, hospital stay, pathology results and up to two follow‐up visits with clinical/biological assessments. Data has been audited in Sweden and Switzerland, and found to be valid and reliable. All participating centers were notified 3 months prior to the extraction of the database and agreed to complete their data collection.
